# Enhancement of Oil Palm Waste Nanoparticles on the Properties and Characterization of Hybrid Plywood Biocomposites

**DOI:** 10.3390/polym12051007

**Published:** 2020-04-27

**Authors:** Arif Nuryawan, C. K. Abdullah, Che Mohamad Hazwan, N. G. Olaiya, Esam Bashir Yahya, Iwan Risnasari, Nanang Masruchin, M. S. Baharudin, Hasmawi Khalid, H. P. S. Abdul Khalil

**Affiliations:** 1 Department of Forest Products Technology, Faculty of Forestry, Universitas Sumatera Utara, Medan 20155, Indonesia; arif5@usu.ac.id; 2 School of Industrial Technology, Universiti Sains Malaysia, Penang 11800, Malaysia; iwemocha@gmail.com (C.M.H.); ngolaiya@futa.edu.ng (N.G.O.); essam912013@gmail.com (E.B.Y.); mohdsyukri@usm.my (M.S.B.); 3 Research Centre for Biomaterials, Indonesian Institute of Sciences (LIPI), JI.Raya Bogor KM 46, Cibinong 16911, Indonesia; masruchin@biomaterial.lipi.go.id; 4 Politeknik Sultan Salahuddin Abdul Aziz Shah, Persiaran usahawan, Seksyen U1, Shah Alam 40150, Malaysia; Hasmawiky@gmail.com

**Keywords:** nanoparticle, hybrid, plywood, mechanical, palm fruit fibre

## Abstract

Using oil palm trunk (OPT) layered with empty fruit bunch (EFB), so-called hybrid plywood enhanced with palm oil ash nanoparticles, with phenol-formaldehyde (PF) resin as a binder, was produced in this study. The phenol-formaldehyde (PF) resins filled with different loading of oil palm ash (OPA) nanoparticles were prepared and used as glue for layers of the oil palm trunk (OPT) veneer and empty fruit bunch fibre mat. The resulting hybrid plywood produced was characterised. The physical, mechanical, thermal, and morphological properties of the hybrid plywood panels were investigated. The results obtained showed that the presence of OPA nanoparticles significantly affected the physical, mechanical, and thermal properties of the plywood panels. Significant improvements in dimension from water absorption and thickness swelling experiments were obtained for the plywood panels with the highest OPA nanoparticles loading in PF resin. The mechanical properties indicated that plywood composites showed improvement in flexural, shear, and impact properties until a certain loading of OPA nanoparticles in PF resin. Fracture surface morphology also showed the effectiveness of OPA nanoparticles in the reduction of layer breakage due to force and stress distribution. The thermal stability performance showed that PF filled OPA nanoparticles contributed to the thermal stability of the plywood panels. Therefore, the results obtained in this study showed that OPA nanoparticles certainly improved the characteristic of the hybrid plywood.

## 1. Introduction

Biocomposites are defined as composites materials that consist of biodegradable matrix and biodegradable lignocellulosic fibre such as oil palm biomass, rice husk, coconut fibre, kenaf, and so on as reinforcement. Furthermore, due to the continuous depletion of bioresources and environmental deterioration, tremendous interest has been drawn to utilize other renewable and sustainable materials [1]. The development of biocomposites with new features and functions has attracted significant attention due to their benefits in environmental and performance properties. Recent developments by Fu et al. [2] in polymer chemistry and nanotechnology reported that the fabrication of hybrid wood materials has great potential for engineering purposes. Useful functionalization approaches for designing biocomposite applications inspired by wood-based materials have made it possible to be used in a structural and non-structural application.

A structural biocomposite can be defined as a product that can carry and hold a load in use. For example, wall panels, stairs, roof structures, and flooring systems in a building are applications of structural bio-composites. These applications are the results of the development of bio-based wood structures by combining load-bearing and functional properties. Moreover, their feasible and competitive mechanical properties have made the biodegradable natural fibre a preferred engineering material for reinforcement [3]. Meanwhile, with the high demand for wide range applications, the manufacturer also produces low-performance materials known as non-structural biocomposites that are not needed to carry a load in use. Therefore, with the rapid expansion of biocomposite applications, the lignocellulosic biomass materials have been given priority to be considered as reinforcement material in thermoplastic and thermoset matrix [1,4]

In wood-based industries, plywood composites are one of the most important types of conventional composites that still relevant today. The plywood name was originated from the wood product where three or more plies of thin sheets of wood and well known wood veneers were bonded together with the application of adhesives. Most of the applications of plywood composites that are relevant until today are due to some of the plywood characteristics that are superior and comparable to solid wood properties. Many applications of plywood, such as in furniture products, structural and non-structural panels, interior and exterior design, have some advantages in terms of cost, processing, design, and so forth. In plywood manufacturing, there are two classes of plywood, which (1) are for construction and industrial, and (2) are for hardwood and decorative plywood [5]. In construction applications, plywood becomes one of the favourable building materials due to the properties of plywood that can easily absorb and release moisture, resulting in the reduction of heat conditions during climatic changes. Furthermore, its ability to withstand large cracking forces makes it suitable for bracing walls [6].

In the past, solid wood has been chosen to produce structural materials due to the excellent properties, but the escalated demand for solid wood has caused the reduction of forest resources. Hence, a plywood product which originated from veneer that was manufactured from good quality timber has been developed in straight, large diameter, and cylindrical forms, as an alternative to solid wood products [7]. As the properties of both solid wood and plywood are comparable, the plywood becomes alternative products to overcome the solid wood shortage. The properties of plywood were affected by the density of the wood, trees species, type of adhesives, thickness of the veneer, number of plies, cured temperature, and pressing pressure [8,9].

In the past few decades, oil palm trunk has been partially utilized to be an alternative supply for wood-based industries and the most viable alternative in plywood production. Even though oil palm trunk is available in a great amount, the successful utilization of oil palm trunk is still limited due to the weaknesses such as being very hygroscopic, a higher rate of shrinking and swelling compared to solid wood. Furthermore, lower strength, poor durability, dimensional stability, and machining properties are the reason for the limited utilization of oil palm trunk [10,11]. However, with the similarities of some properties as compared to solid wood, the outer part of the oil palm trunk has been chosen and the most suitable part for this purpose. This because of the composition of the oil palm trunk that contains more vascular bundles in the outer part, as the centre part contains soft parenchyma tissue [12].

The utilisation of oil palm trunk veneer to produce plywood has been successfully reported by Mokhtar et al. [10]. Hardwood was used as the face and back of the plywood. Currently, in plywood making, oil palm veneer is primarily used as an inner or core layer to reduce the use of solid wood veneer. Solid wood veneer is subsequently being applied as a face and back layer to produce comparable properties as compared to plywood panels from ordinary wood. In addition, due to the soft structure of the oil palm veneer, the veneer is only suitable as a core layer integrated with a face and back layers of hardwood. A recent study by Masseat et al. [13] reported that the properties of oil palm lumber or veneer are inferior as compared to ordinary wood. To enhance the quality and dimensional stability, modification, and treatment of the oil palm tree are required. By using oil palm veneer as a core layer, it is sufficient to provide strong stability and utilise in short term applications such as packaging material and non-structural design. Mass utilization of oil palm trunk veneers as raw material to form value-added products could reduce the environmental burden and significant role in sustaining natural resources [14].

Since oil palm trunk is a potential raw material for value-added products, particularly in plywood industries, it is possible to utilize it with other lignocellulosic materials to produce better products to meet market demand [15]. Moreover, with the declining supply of timber and wood materials, the hybrid plywood could help to reduce the environmental burden of wood consumption. Several studies have been investigated the production of hybrid plywood to enhance the properties of the lignocellulosic composite, such as greater deformability, less abrasiveness to machining properties, improve biodegradability and lower the production cost as mention by Abdul Khalil et al. [6].

Researchers have reported the use of adhesives as glue in plywood [15,16]. Bardak et al. reported the significant improvement of bonding strength due to the addition of nano-TiO_2_ into the urea-formaldehyde (UF) matrix [17]. The increased bonding strength and screw withdrawal resistance also reported by Candan and Akbulut [18] due to the addition of nano-SiO_2_ into UF adhesives. Findings by Xu et al. [19] showed that the modification of soy protein adhesives with Nano-SiO_2_ obtained more significant interaction between adhesives and wood plies. This behaviour related to the elimination of surface voids by Nano-SiO_2_ thus develops higher interfacial interaction between adhesives and wood plies. As reported by Dukarska and Czarnecki [20] in manufacturing water-resistant plywood, the addition of nano-fumed silica into melamine urea phenol formaldehyde (MUPF) showed significant improvement in moisture resistance due to the increase of activation energy and cross-linking process of MUPF. According to Zhang et al. [21], the addition of alumina nanoparticles as additives in PF resin significantly enhances the wet shear strength of plywood as a result of the curing process optimization and PF resin bonding strength.

At present, many researchers have studied the utilization of oil palm trunk veneer to produce hybrid plywood with appropriate production technologies such as hybrid bamboo mat-wood veneer [22], bamboo strip-oil palm wood veneer [23,24] and empty fruit bunch (EFB) fibre mat-oil palm wood veneer [25,26,27]. A recent study by Ashori et al. [27] reported that the use of waste tire rubber (WTR) in hybrid plywood as a ply enhances the mechanical properties, reduces thickness swelling and water absorption in plywood composites. Based on the recent study in the utilisation of oil palm trunk as veneer raw material for plywood, to produce superior plywood products, the different types of material characteristics can be used, such as fibrous layers, clad metal, fibreglass and so forth. Moreover, the application with a different type of resin with good bonding properties into layers also can produce plywood as strong as ordinary wood [28]. Hence, this plywood composite with significant characteristics will be used in the design of the high-performance structure and be able to meet market demands in the future.

## 2. Materials and Methods

### 2.1. Material

Oil palm ash (OPA) was obtained from United Oil Palm Mill, Penang, Malaysia. Empty fruit bunch (EFB) fibre mat was obtained from Ecofibre Technology Sdn. Bhd., Penang, Malaysia. The oil palm trunk (OPT) veneer was supplied by Emas Cergas Express Enterprise, Kedah, Malaysia, and phenol-formaldehyde (PF) resin was obtained from Hexion Specialty Chemicals Sdn. Bhd, Penang, Malaysia. In addition, commercial plywood (PWC) was made from seraya timber with a phenol formaldehyde resin binder. The PWC was five (5) layered like the hybrid plywood to be produced and of thickness 10 mm.

### 2.2. Preparation of Oil Palm Ash Nanoparticle

The OPA form the hopper outlet was dried at 70 °C for 24 h in the oven to remove the excess water content. Then the dried OPA was sieved by 60 mesh size sieves to separate micro-sized particles such as sands, stones, and macro-particles from the water tube boiler. The fine dried oil palm ash was further ground to smaller particle size, followed by the optimisation process using high-energy ball milling (horizontal ball milling) and procession parameters stated in Table 1. Then, the nano oil palm ash from the ball milling process was kept at high-temperature conditions around 120 °C in the drying oven for 24 h to prevent agglomeration and was kept in a dry place to avoid contact with moisture.

The particle size of the milled powders was measured with a Malvern Mastersizer (Malvern Instruments Ltd., Grovewood Road, UK) scirocco 2000 by dynamic light scattering measurements. In each measurement, the detector and laser were aligned, and the background was calibrated. Size distribution was quantified as the relative volume of particles in size bands presented as size distribution curves (Malvern MasterSizer Software v 5.60, Grovewood Road, UK).

### 2.3. Preparation of Nanocomposite Binder from Phenol-Formaldehyde (PF) Resin Filled OPA Nanoparticles

The nanoparticle was dried at 105 °C for 24 h and cooled in the desiccator containing silica gel to prevent moisture absorption. A specially prepared stainless-steel mould with dimensions of 120 × 100 × 3 mm was used to fabricate the nanocomposite. Then, the phenol-formaldehyde resin was mixed with the desired amount (2%) of OPA nanoparticles at room temperature for 30 min using a mechanical stirrer at 800 rpm. The 2% of OPA used is required to ensure the homogenous dispersion of OPA nanoparticles in the PF resin matrix [29]. This mixture was then degassed in a vacuum chamber for five minutes to remove bubbles. The mixture was then cast into the mould, and the polymerization process was left to cure in the oven at 150 °C for 30 min [30]. Finally, once the nanocomposite was cured, the nanocomposite was removed from the mould and left in the conditioning room for 24 h.

### 2.4. Preparation of Hybrid Plywood Composites

**Figure 1:**
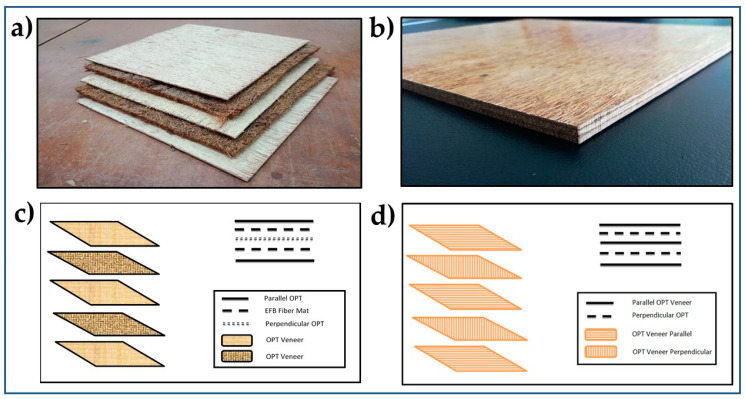
(**a**) Layering pattern of five plywood hybrid, (**b**) hybrid plywood composite, (**c**) schematic arrangement of hybrid plywood, (**d**) schematic arrangement of OPT veneer plywood.

The preparation of OPT veneer and EFB fibre mats were shown in Table 2. For the development of composite, a stainless-steel plate and thickness bar with dimensions of 320 × 320 × 10 mm were used. Hybrid plywood composites were developed by using hand lay-up techniques for making a test sample. Layering pattern of 5-ply hybrid plywood and the final dimension of hybrid plywood composite was shown in Figure 1a,b. Schematic arrangement of 5-ply OPT veneer and EFB mat to produce plywood and hybrid plywood were shown in Figure 1.

The layers were glued using phenol-formaldehyde (PF) resin filled OPA nanoparticles with a glue spread rate of 400 g/m^2^ before being cold-pressed for 10 min. The choice of using 400 g/m^2^ was due to the characteristic of the OPT veneer and EFB fibre mat (Bekhta [31]). Preliminary studies using different level glue spread (200, 300, 400, 500, 600 g/m^2^) showed that the performance of plywood composite significantly deteriorated in lower level glue spread due to poor layer bonding [32]. Meanwhile, with the higher level of glue spread, the brittleness of plywood composite increased, resulting in mechanical strength reduction. As reported by Bekhta and Marutzky [32], the effective usage of glue is much dependent on the ply’s characteristics such as surface roughness and required thickness to create a continuous glue layer of the plywood composites. The spread level for the individual layer was calculated based on Equation (1).

(1) $$Spread\;Level\;\left(400\;gm^{-2}\right)=\frac{\mathrm{Weight}\;\mathrm{of}\;\mathrm{the}\;\mathrm{resin},\;g}{\mathrm{Length}\;\times \;\mathrm{Width}\;\mathrm{of}\;\mathrm{veneer},m^{2}}$$

The cold press method is intended to develop consolidation between the surfaces of the raw materials. After that, they were then hot-pressed for 30 min at a temperature of 150 °C for phenol-formaldehyde, at approximately 200 bars (3000 psi) of pressure.

### 2.5. Characterization of Phenol-Formaldehyde Nanocomposite Filled OPA Nanoparticles and Plywood Panels

#### 2.5.1. Physical Properties

● Moisture Content Measurement

The determination of the moisture content of each test sample was done using American society for testing and materials ASTM D1037 Standard. The weight of each test sample before the time of sampling and its state after drying to constant mass at 103 ± 2 °C was determined. The weight of each test sample was recorded to an accuracy of 0.01 g by using an analytical balance. The test pieces were placed in the drying oven at a temperature of 103 ± 2 °C until the constant mass has been reached. The test samples were then cooled to approximately room temperature in the desiccator, and the weight of each test sample to an accuracy of 0.01 g was recorded. The moisture content, H, of each test samples was determined as a percentage by mass to the nearest 0.1% using Equation (2)

(2) $$Moisture\;Content\;\left(\%\right)=\frac{W_{2}-W_{1}\;}{W_{1}}\;\times \;100$$

where *W_2_* is the initial weight of the sample, and *W_1_* is the final weight after the oven-dry.

● Density Profile Characterization

The density profile of the samples along the thickness of panels was determined using Density Profiler Grecon model DA-X (Alfeld, Germany). The samples with a dimension of 50 × 50 × 10 mm were cut and maintained in a chamber at ambient temperature (25 ± 3 °C) and relative humidity of 30% (±2%) before testing. During scanning, the samples were inserted into the cassette holder for each batch scan and analyzed every 0.025 mm/s. In each case, the density profiles of the five samples were tested, and the average value is tabulated.

● Water Absorption and Thickness Swelling

The water absorption behaviour and the effect of thickness swelling on samples (50 × 50 mm) due to absorbed water were determined using BS 317:1993 Standard. The panels were submerged in clean water at (20 ± 1 °C), having a pH of (7 ± 1). The water absorption and thickness swelling were measured by the difference in weight and thickness of the samples, respectively, before and after 24 h immersion in water. The water absorption (Equation (3)) and thickness swelling (Equation (4)) of the samples were calculated as percentages and are measured by using electrical balance and digital slide calliper, respectively. The water absorption and thickness swelling test was continued for several days (up to 10 days) until the constant weight and thickness of the samples were obtained.

(3) $$Water\;Absorption\;\left(\%\right)=\frac{M_{2}-M_{1}\;}{M_{1}}\;\times \;100$$

where *M_1_* is the weight of the test piece before immersion, in grams, and *M_2_* is the weight of the test piece after immersion, in grams.

(4) $$Thickness\;Swelling\;\left(\%\right)=\frac{T_{2}-T_{1}\;}{T_{1}}\;\times \;100$$

where *T_1_* is the thickness of the test piece before immersion, in millimetres, and *T_2_* is the thickness of the test piece after immersion in millimetres.

#### 2.5.2. Mechanical Properties

● Flexural Test

The modulus of rupture (MOR) and modulus of elasticity (MOE) in the flexural test was determined by applying a load to the centre of a test piece supported at two points. The flexural test was performed according to BS 310:1993 standard using an Instron Model 5528 Testing Machine with 100 KN maximum loading. The bending test was carried out using rectangular strips with dimensions of 250 × 50 × 10 mm. Samples were tested at a crosshead speed of 6 mm/min and a support span of 240 mm. All the specimens were conditioned at an ambient temperature of 25 ± 3 °C and relative humidity of 30% (±2%) before testing.

● Shear Test

The shear strength of the plywood panels was examined according to British Standard EN 314-1 and EN 314-2 for the plywood bonding class 2 (humid conditions). The plywood panels were conditioned for at least one week under laboratory conditions before being cut into ten pieces with dimensions of 100 × 25 mm (glued area of 25 × 25 mm). The specimens were immersed in boiling water for 6 h, removed from the water, and then cooled in the water at a temperature of 20 ± 3 °C for at least 1 h before the shear strength measurement. The shear strength data were reported and represent the average of ten samples for each plywood batch type.

● Impact Test

The Izod notched impact test was performed using the Go-Tech testing machine, Model GT-7045 MD. Izod impact test was carried out according to the ASTM D256 standard. The sample of the impact test was prepared with a dimension of 70 × 15 × 10 mm. Before the test was done, V-notch was made on the sample by using the Go-Tech V-notch machine. The depth of V-notch was 2 mm, and the angle of V-notch was 90°. The energy of impact pendulum was 5 J and speed 3.46 m/s. All the specimens were conditioned at ambient temperature (25 ± 3 °C) and relative humidity of 30% (±2%) before testing.

#### 2.5.3. Thermogravimetric Analysis (TGA)

A Perkin Elmer thermal gravimetric analyzer (TGA-6) was used to investigate the thermal stability of the plywood panels applied with PF resin-filled OPA nanoparticles. The investigation of the thermal stability was analysed in powder form approximately 5–7 mg/analysis. The powder form of samples was heated from 30 to 900 °C under the nitrogen atmosphere at a heating rate of 20 °C/min. The results of the TGA simultaneously recorded the percentage weight loss of the materials.

#### 2.5.4. Morphological Properties

● Transmission Electron Microscopy (TEM)

Transmission electron microscopy (TEM) was carried out with an EFTEM Libra–Carl Zeiss instrument; the OPA nanoparticles were oven-dried before using the TEM to characterize the size and morphology of the particles. The OPA nanoparticles were prepared in acetone and dispersed with an ultrasonicator for 10 min. The samples for TEM analysis were obtained by placing a drop of colloidal dispersion containing OPA nanoparticles onto a carbon-coated copper grid. Embedded OPA nanoparticles were air-dried at room temperature before being examined under TEM instrument under control condition.

● Field Emission Scanning Electron Microscopy (FESEM)

Field emission scanning electron microscope FESEM-Carl Zeiss from Unit of Microscope Electron, Centre of Archeology Global Research, Universiti Sains Malaysia, Penang was used to examine the morphological images of phenol-formaldehyde (PF) resin nanocomposite filled OPA nanoparticles and plywood panels applied with PF resin-filled OPA nanoparticles. A thin section of the sample was mounted on an aluminium stub and was sputter-coated with gold before enhancing morphological examination. The FESEM micrographs were obtained under conventional secondary electron imaging conditions with an acceleration voltage of 5 kV.

## 3. Result and Discussion

### 3.1. Particle Size Distribution Palm Oil Ash

**Figure 2:**
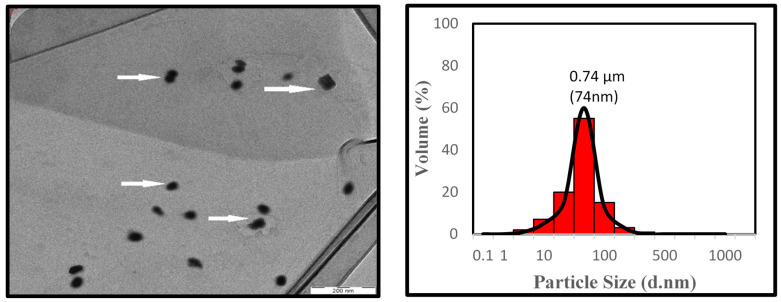
TEM micrograph and particle size distribution graph of oil palm ash nanoparticles.

Figure 2 shows the TEM image of OPA nanoparticles, which is by particle size distribution, which was evaluated by laser diffraction particle size analyzer. From the TEM image, the OPA nanoparticles appear in black. The black colour was probably due to that less transparent towards electron beam and electronic density of the OPA nanoparticles element, as reported by Pishvaei and Farshchi [33] and Yazdimamaghani et al. [34]. The TEM micrograph reveals that the OPA nanoparticles possessed irregular size and were uniformly distributed within the 70 to 200 nm range without having agglomeration. This similar finding also reported by Abdul Khalil et al. [35].

### 3.2. Properties of EFB Fibre Mat/OPT Veneer Hybrid Plywood

#### 3.2.1. Physical Properties

● Moisture Content

**Figure 3:**
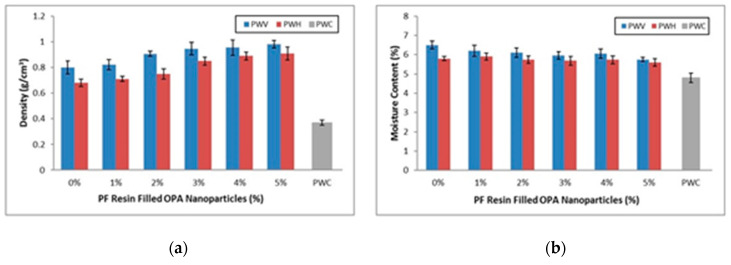
(**a**) Density profile and (**b**) moisture content of plywood veneer (PWV), hybrid plywood (PWH), and commercial plywood (PWC) with different loading of phenol-formaldehyde (PF) filled OPA nanoparticles.

The moisture content of plywood veneer (PWV), hybrid plywood (PWH), and commercial plywood (PWC) are shown in Figure 3a. From the results, it showed that the plywood composites exhibited average moisture content in a range of 5% to 7%. It can be observed that PWH showed slightly lower moisture content compared to PWV and comparable with PWC.

The issue of the hydrophilic nature of the EFB fibre mat is well known. The presence of PF resin uniformly spread on the fibre has facilitated the interface bonding, which reduces the moisture absorption capability and improves mechanical properties [36,37]. As for PWH and PWV, respectively, the moisture content slightly reduces with the increase of OPA nanoparticles. It is indicated that the PWH and PWV with PF filled 5% OPA nanoparticles showed the lowest moisture content compared to other plywood composites. However, the lowest moisture content was still exhibited by PWC due to materials from solid wood that initially had lower moisture content compared to OPT veneer and EFB fibre mat. The PWV showed higher moisture content compared to PWH and PWC, probably due to parenchyma tissue comprises in OPT veneer. In nature, the parenchyma tissue behaves like a sponge and is prone to absorb moisture. Even though the OPT veneer was taken from the outer part of the OPT, which has the lowest volume of parenchyma tissue, it still could hold high moisture content due to hygroscopic characteristics.

Meanwhile, the higher moisture content in lower loading of OPA nanoparticles in PWH and PWV may be due to PF resin that formed macro and micro void after curing. This behaviour will cause moisture incorporated with the porosities resulting in more absorption of moisture from the air. Thus, the accumulation of moisture leads to higher moisture content and further potentially decrease mechanical properties and accelerate decay deterioration [38].

● Density Profile

Figure 3b shows the density of plywood veneer (PWV), hybrid plywood (PWH), and commercial plywood (PWC). The figure illustrated that the density of PWV and PWH increased radially with the increase of OPA nanoparticles loading. In the meantime, PWV and PWH showed the highest density at 5% OPA nanoparticle loading. However, both PWV and PWH still obtained a lower density compared to PWC. It is observed that increasing density for both PWV and PWH due to higher loading of OPA nanoparticles inside the PF resin. When plywood was hot-pressed under the condition of high temperature, the compressive stress was imparted to the veneer microstructure leaving a hollow space between vascular bundles and parenchyma tissues. This explained that PF resin-filled OPA nanoparticles applied on PWV were homogeneously spread on the surface of OPT veneer, occupied the hollow space between the vascular bundles, and thus obtained better PWV composite with great density.

**Figure 4:**
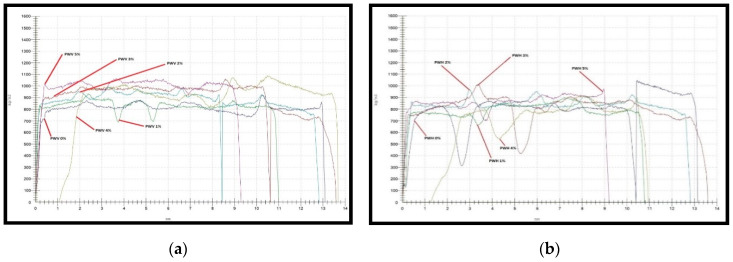
Density profile of (**a**) PWV and (**b**) PWH with different loading of PF resin-filled oil palm (OP) nanoparticles.

PWH also exhibited higher density at 5% loading due to OPA nanoparticles themselves. Feng et al. [12] reported that filler helps to control the flow of the resin on the veneer surface and between the fibres. The energy provides by OPA nanoparticles assists the resin in penetrating within the veneer and fibre, resulting in higher density obtained for hybrid plywood (PWH). Previous work by Robertson et al. [39] examined the influence of filler in the plywood. It stated that the presence of filler in the resin or adhesives controlled the spread rate, uniformity of resin flow and enhanced the viscosity stability, which is reflected by the increase of density on each PWV and PWH with different loading PF filled OPA nanoparticles. In the meantime, both PWV and PWH exhibited higher density compared to PWC, as shown in Figure 4. This was due to OPT veneer itself that had a higher density compared to veneer in commercial plywood. In addition, most of the commercial plywood (PWC) used light hardwood as a veneer that had a density in the range 0.4–0.7 g cm^−3^. The lower density of commercial plywood also could be explained by cell structures. A typical hardwood to produce veneer and plywood such as meranti has a bigger lumen diameter of 15.4 µm [40] compare to oil palm trunk in range 9–11 µm [41]. As density is obtained by dividing the weight (in gram) by its volume (cubic centimetre), thus greater volume contributed by cell structures in wood veneer, then the lower density of plywood will be obtained.

The density distribution of PWV and PWH is shown in Figure 4a,b, respectively. The PWV (5%) showed the highest and uniformity of density compared to other PWV. The evenness of PWV density was due to veneer bonding that reported by Luo et al. [42]. The report explained that resin permeates into gaps and porous space between veneers then cured to form an interlock between veneer layers. Therefore, the graph in Figure 4a showed a very small width between peak and consistency of peak altitude of PWV in higher PF resin-filled OPA nanoparticles. Meanwhile, PWV (0%) and PWV (1%) showed low readings of density, respectively. This was due to the lower content of OPA nanoparticles that could assist the resin in permeating into the veneer, thus help to increase the density of PWV.

Figure 4b illustrated the density distribution of PWH. From Figure 4b, PWH (5%) exhibited showed the stability of peak altitude and peak width that contributes to higher density reading. In hybrid plywood, the gap and porous space between veneer and fibre mat were wider. The presence of OPA nanoparticles in the PF resin helped to fill the space between the veneer and fibre mat. The density of PWH (5%) increased but the density obtained from the density profiler was still low compared to PWV (5%). Meanwhile, lower density obtained by PWH (0%) and PWH (1%) was due to a higher volume of void space that unoccupied after the PF resin cured and harden between the veneer and fibre mat. The variation of peak width and altitude of PWH (1%) indicated that lower loading of PF filled OPA nanoparticles diminished the penetration of PF resin into porous space and certainly reduced the density of PWH. An investigation by He et al. [43] and Zeng et al. [44] found out that irregular resin distribution on the veneer would significantly decrease the density due to limited interphase bonding between veneer and fibre mat after the resin cured.

#### 3.2.2. Water Absorption Properties

**Figure 5:**
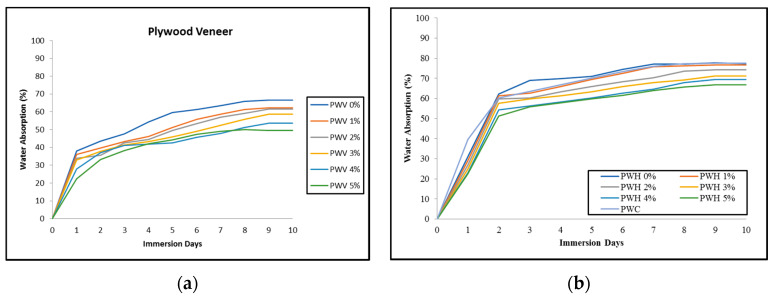
(**a**) Water absorption (%) of PWV and (**b**) water absorption (%) of PWH with different loading of PF filled OPA nanoparticles.

The water absorption behaviour of plywood veneer (PWV) is shown in Figure 5a,b. Understanding of water absorption properties is essential for plywood to improve dimensional stability. Research findings stated that the higher the value of water absorption, the weaker the wood-based panel could be. Several factors can influence the properties of water absorption, such as fibre loading, area of the exposed surfaces, diffusivity, void content, etc. The PWV of each PF resin-filled OPA nanoparticle loading showed the significant value of water absorption, although their value was slightly closure compared to each other. The results indicate that PWV (5%) obtained the lowest value of water absorption followed by PWV (4%), PWV (3%), PWV (2%), PWV (1%) and PWV (0%) after 10 days of water immersion.

From the water absorption curve in Figure 5a, PWV (0%) obtained the highest water intake, which up to 60%. This behaviour could be the effect of the hydrophilic nature of OPT veneer that consists of parenchyma tissue, which tends to absorb moisture, especially water. Moreover, lower loading of PF filled OPA nanoparticles could cause water easily permeates into OPT veneer structures, resulting in more water penetration in PWV (0%). As the loading of PF resin-filled OPA nanoparticles increased, the amount of water absorption of PWV decreased significantly. The lower water intake into PWH (5%) is mainly attributed to less void or porous area that has been occupied by OPA nanoparticles. As reported by Cai et al. [45], the cured resin occupied and remained in the cell wall and lumen of wood structures, thus shielding the wood surface from water penetration into the wood. Other reasons for the decrease of water absorption are probably due to the resin that might react with the numerous hydroxyl group contained in the OPT veneer component certainly contribute to the reduction of water absorption [46]. Research by Abdul Khalil et al. [6] stated that phenol-formaldehyde resin provides greater moisture stability to plywood due reaction in the cross-linking process, which hydroxyl group will absorb moisture or water through the formation of hydrogen bonds.

Results of water absorption of hybrid plywood (PWH) with different loading of PF resin-filled OPA nanoparticles compared to commercial plywood (PWC) are shown in Figure 5b. It could be observed that water absorption uptakes for PWH were much higher compare to PWV. This behaviour showed that the hydrophilic properties of EFB fibre responsible for water absorption in the composites. Besides, weak interaction between the EFB fibre and OPT veneer could lead to the formation of void microstructures within the PWH, which facilitates water absorption. Particularly, the increases in concentration of PF filled OPA nanoparticles in the PWH greatly reduced the water absorption. The application of PF resin as a binder assisted the EFB fibre in impeding the permeation of water molecules into cell wall structures, thus decreasing the degree of water absorption.

Meanwhile, OPA nanoparticles with help from the PF resin viscosity would impregnate into the void and unoccupied microstructures area to block the entrance of water molecules. This might be attributed to the water-resistant of plywood composites. Sultan et al. [47] reported that water uptake value was influenced by the behaviour of the nanofiller itself, which with the capability of the nanofiller to interact with water molecules, thus contributed to increase or decrease water absorption values.

#### 3.2.3. Thickness Swelling Properties

**Figure 6:**
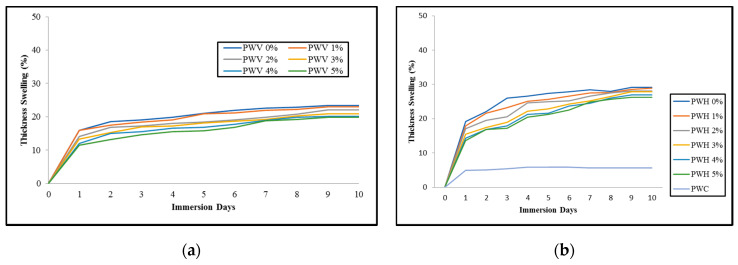
Thickness swelling (%) of **a**) PWV and **b**) PWH with different loading of PF resin-filled OPA nanoparticles.

The thickness swelling of plywood veneer (PWV), hybrid plywood (PWH), and commercial plywood (PWC) after being submerged in water for 10 days, reaching a maximum value for which no more dimensional changes are shown in Figure 6a,b respectively. Generally, the thickness swelling increased directly proportional to immersion time until the equilibrium phase was achieved. Principally, the rates of thickness swelling of PWV, PWH, and PWC were low during the initial stages of moisture absorption. This is probably due to the moisture absorption process until the cell wall was saturated with water, and the excess water existed as free water that could lead to composite delamination [27,48]. In general, the thickness swelling of PWV illustrated in Figure 4. showed that the PWV with the highest loading of PF filled OPA nanoparticles exhibited the lowest percentage as compared to other PWV composites. The results indicated that with the increase of PF filled OPA nanoparticle loading, the thickness swelling significantly decreased. The thickness swelling (TS) is an important parameter for composite. Generally, the swelling rates for polymer matrix compos ites are low during the initial stages of moisture absorption due to the viscoelastic ity of the polymer matrix. In addition, any pores or voids that are present after fabrication will help to accommodate some of the swellings of the added wood.

The decrease in thickness swelling is mainly due to the concentration of OPA nanoparticles that act to control the permeation of moisture, thus stabilizing the dimensional stability of the PWH. Research by Azzam [49] demonstrates that nanofiller could work effectively as a swelling reducer. In addition, the PF filled OPA nanoparticles that occupied the void area could assist in stabilizing the swelling process until relative isotropic behaviour to both diametrical and axial aspect. The application of PF resin-filled OPA nanoparticles as a binder in plywood composites resulted in decreased void space and the porous area, thus reducing the swelling capacity of PWV and PWH. This finding is similar to Leemon et al. [50], who concluded that the PF resin-filled nanofiller could occupy the void and porous area effectively due to the crosslinking monomer in intercellular cell and consequently reduce the thickness swelling of the composites.

Similar trends of thickness swelling PWV also were shown in Figure 6b. It is observed that the addition of OPA nanoparticles into PF resin significantly reduced the thickness swelling. The PWH with the highest PF filled OPA nanoparticles showed the lowest value of thickness swelling compared to other PWH composites. Meanwhile, plywood commercial (PWC) exhibited the lowest value of thickness swelling as compared to PWV and PWH. This is because the PWC has better physio-chemical properties as compared to PWV and PWH composites, mainly due to the capillaries in wood, which is one of the structures to control the dimensional changes of woods.

In comparison, the higher thickness swelling in PWH is mainly attributed to the hydrogen bonding of the moisture to the free hydroxyl groups that are present in the cellulosic fibre. Therefore, the diffusion of water molecules into PWH was higher compared to PWV. These results were in good agreement with a study performed by Ashori et al. [27].

The PF filled OPA nanoparticles could illustrate crystal structures in the form of nanocomposites. There would be a possible reason that the change in the degree of crystallinity could reduce the swelling process of the composites [51]. It can be observed that the highest PF filled OPA nanoparticles, both for PWV and PWH composites, revealed the lowest value of thickness swelling. After the PF resin cured and hardened, the crystalline regions were established and reduced the permeability of the moisture. This behaviour could lead to enhance barrier properties and reduce swelling behaviour of the plywood composites. As for neat PF resin, the cured resin is prone to cracking due to the PF resin brittleness characteristic, and this phenomenon allows water to penetrate the bonded layers resulting in higher swelling of plywood composites. Therefore, the application of PF resin-filled OPA nanoparticles for producing PWV and PWH improved the dimensional stability significantly as compared to the pure PF resin.

#### 3.2.4. Mechanical Properties

● Flexural Strength

**Figure 7:**
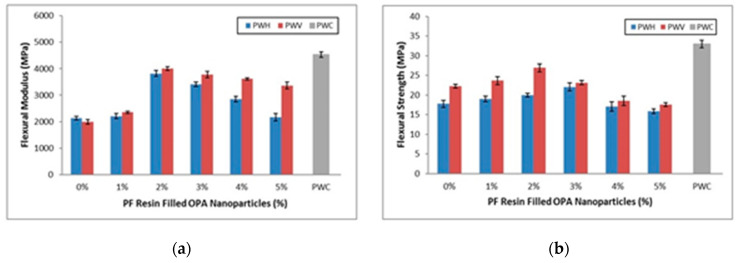
(**a**) Flexural modulus and (**b**) flexural strength of PWV and PWH with different loading of PF filled OPA nanoparticles compared to PWC.

In general, flexural properties will determine the strength (MOR) and rate of deflection, known as modulus of elasticity (MOE) of the composites. These characteristics are the most important parameters to refer to for engineering purposes. The flexural properties of plywood veneer (PWV) and hybrid plywood (PWH) as compared to commercial plywood (PWC) are shown in Figure 7a. Based on the figure, the PWV with 2% of PF resin-filled OPA nanoparticles showed the greatest as compared to other PWV and PWH. Meanwhile, PWH with 3% of PF resin-filled OPA nanoparticles exhibited the highest strength as compared to other PWH. From the figure, it can also be observed that, after PWV and PWH composites achieved the highest strength at 2% and 3% PF resin-filled OPA nanoparticles, the flexural strength started to decrease.

At the higher loading of PF resin-filled OPA nanoparticles, 4%, and 5% respectively, the flexural strength decreased and showed slightly different PWV and PWH composites. Although there was an improvement of flexural strength on PWV and PWV composites after using PF resin-filled OPA nanoparticles as a binder, the commercial plywood still exhibited the highest strength (33 MPa). During the flexural test, the PWV and PWH composites were not completely broken after reaching the maximum load. This behaviour is probably due to the characteristic of the material, which is OPT veneer and EFB fibre mat that has greater extensibility to stand on load given. However, the failure of the PWV and PWH occurred when the matrix/binder started to crack and propagate between the layers. Similar results were also obtained by Jawaid et al. [52] that the hybrid composites using natural fibre as reinforcement were completely unbroken at peak load.

Moreover, the decrease of flexural strength is probably due to the irregular distribution of OPA nanoparticles in PF resin during the preparation process, which causes concentration due to particle agglomeration. Other reasons may be due to untreated OPA nanoparticles and poor interfacial bonding between PF resin, OPA nanoparticles, and plywood panels resulting in a reduction of PWV and PWH flexural strength. Hence, it is obvious that the application of PF resin-filled OPA nanoparticles as a binder affected the flexural performance of both PWV and PWH composites.

The flexural strength of PWV slowly increased as the PF resin-filled OPA nanoparticle loading increased from 1% to 2%. This finding indicates that the presence of OPA nanoparticles in resin up to optimum loading allowed the PWV to stand the maximum force given. When the loading of PF resin-filled OPA nanoparticles rose from 3% to 5%, the strength of the PWV began to decline. It is assumed that the decline of flexural strength of PWV with PF resin OPA nanoparticles beyond 2% loading is due to excessive aggregation of OPA nanoparticles embedded in PF resin. The evolvements of OPA nanoparticles aggregation in the PWH composites reduced the ability to disseminate the force between PF resin and OPT veneer. This behaviour may negatively affect the performance of PWV flexural strength properties [53].

● Flexural Modulus

Figure 7b showed the effect of different loading PF resin-filled OPA nanoparticles on the flexural modulus of PWV, PWH, and PWC. Many researchers have discovered that the use of a filler with a certain type, size, and shape leads to greater improvement in mechanical properties [54]. From the graph, it is clearly explained that the flexural modulus of specimens shows an identical trend with the flexural strength. There was an initial increase of modulus of elasticity and then decreased with increasing of PF resin-filled OPA nanoparticles loading more than 2%. Despite the decline of the flexural modulus of both PWV and PWH, respectively, the presence of OPA nanoparticles in the PF resin still assisted the specimens in holding the greater load.

A similar trend was also discovered by Abdul Khalil et al. [55], that with the addition of OPA nanoparticles above 3%, there was a reduction in flexural modulus. This phenomenon may be due to OPA nanoparticle agglomeration at higher loading in PF resin resulting in non-uniform dispersion between surfaces and hence deteriorating their flexural modulus. According to Karaman et al. [56] and Ismaeilimoghadam et al. [57] in their research, the modulus of elasticity (MOE) of specimens also increases with the addition of nanoparticles from 0% to 3%. However, the addition of higher content of nanoparticles more than the optimum level certainly decreases the modulus properties. The significant reduction of flexural modulus may relate to OPA nanoparticle agglomeration and aggregation at the end of the curing process.

It was observed that the flexural modulus of PWV obtained a higher value than PWH regardless of the difference of PF resin-filled OPA nanoparticles loading. This is probably because of the smoother surface of PWV that helps the PF resin-filled OPA nanoparticles disperse homogenously, and therefore the stress is efficiently transferred between surfaces. Meanwhile, the lower flexural modulus obtained by PWH compared to PWV was probably due to improper bonding between EFB fibre and plywood veneer. This issue was found out by Deka and Maji [58], that the presence of nanoparticles may reduce the moisturizing ability of lignocellulosic fibre, and the matrix then leads to a significant reduction of flexural properties.

From Figure 7b, it was also shown that the flexural modulus of PWV slightly decreased with the addition of PF filled OPA nanoparticles loading of more than 2% compared to PWH. The flexural modulus of the PWV and PWH were largely depended on the dispersion of OPA nanoparticles in PF resin. Higher content of OPA nanoparticles increased the viscosity of the matrix that slowed down the PF resin spreading process to the whole surface of the EFB fibre mat and plywood-veneer. Nevertheless, the smoother surface of plywood veneer helped the PF resin easily flow with lower hindrance compared to the EFB fibre mat that had multiple layers of fibre. Therefore, the flexural modulus of PWV showed a slight reduction compared to PWH after a particular point of PF resin-filled OPA nanoparticles (2%). The effects of nanoparticle dispersion in subtracting on the flexural modulus were examined by [59], and [57] also found out that the degree of nanoparticle dispersion in subtracting contributed to the enhancement of the flexural modulus.

The enhancement of PWV and PWH mechanical properties, particularly in flexural properties, was contributed by the OPA nanoparticles that had an excellent characteristic such as vast surface area, high surface energy, and is chemically reactive. Moreover, the OPA nanoparticles could react with active elements of resin or adhesives and improve their performance by the volume of interaction at the interface between PF resin and OPA nanoparticles. The previous study by Murphy [54] also stated that there was a correlation between nanoparticle surface activity and polymer, which could lead to an improvement in mechanical properties. However, the tendency of nanoparticles to agglomerate is higher with the increases of surface energy and resulting reduction of mechanical properties [60].

From Figure 7b, it is understood that due to the increases of PF resin-filled OPA nanoparticles loading into PWV and PWH up to 2%, the flexural modulus improved. This behaviour is probably due to OPA nanoparticles that provide the vast surface area with the increase of PF resin-filled OPA nanoparticles loading. The escalation of the surface area enhanced the bonding between PF resin and nanoparticles, hence contributing to significant stress distribution. Hence, the plywood composites became more rigid and more resistant to rupture. Nevertheless, the reduction of flexural modulus with an increase of PF resin-filled OPA nanoparticles from 3% to 5% might be due to the agglomeration of OPA nanoparticles. An investigation conducted by Candan and Akbulut [53] showed that the flexural modulus of plywood panels was significantly affected by the nanomaterial loading level. It was also reported by Rosamah et al. [61] that the improvement of flexural modulus depended on the nanomaterial loading level and certainly affected the degree of interfacial interaction due to the agglomeration. According to Murphy [54], the number of interactions that developed depended on the surface activity and hydrodynamic effects, which were mainly controlled by the particle size.

● Shear Strength Properties

**Figure 8:**
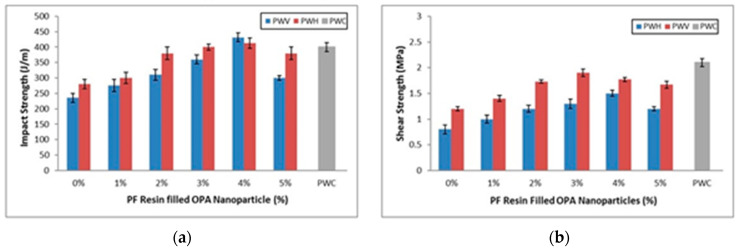
(**a**) Shear strength and (**b**) impact strength of PWV and PWH with different loading of PF filled OPA nanoparticles compared to PWC.

The shear strength values were obtained by the resistance of plywood to deflection caused by shear load [62]. The influences of PF resin-filled OPA nanoparticles on the shear strength of the plywood veneer (PWV), plywood hybrid (PWH), and commercial plywood (PWC) are shown in Figure 8a. The shear strength of PWV increased parallel with the increase of PF resin-filled OPA nanoparticles until a point (3%). Meanwhile, the highest shear strength of PWH was exhibited at 4% of PF resin-filled OPA nanoparticles loading.

Significant enhancement of shear strength showed a similar trend as well as flexural strength and flexural modulus. It is observed that the shear strength increased by increasing the loading of PF resin-filled OPA nanoparticles. Then, the shear strength declined subsequently by higher loading of PF resin-filled OPA nanoparticles. The highest shear strength of PWV at 3% PF resin-filled OPA nanoparticles loading could be attributed to the interpenetrated network, which causes the OPA nanoparticles to fill in the porosity of the plywood veneer during the curing process. Li et al. [63] prove that introducing the filler into a matrix up to a certain level increases the shear strength of plywood.

Furthermore, due to low surface energy, the tendency of OPA nanoparticles to agglomerate becomes lesser. This behaviour assists the OPA nanoparticles in PF resin distributed more homogenously between plywood layers, which further reduce the interior stress. In addition, with the uniform distribution of PF resin-filled OPA nanoparticles, heat transfer during the curing process could ease the release of interior force to be balanced, which could improve the shear strength of plywood.

The results obtained in this present study were supported by the previous study by Luo et al. [42]. As reported by Wang et al. [64], the bonding strength of the plywood panels improved with the addition of nanoclay in PF resin. The presence of nanoclay in the PF resin enhanced the bond line structure of plywood panels and improved the rate of loads transfer distribution homogenously. As can be seen clearly in Figure 8a, the shear strength of PWH subsequently increased with the increment of PF resin-filled OPA nanoparticles up to 4%. Then, the shear strength of PWH slightly degraded, but the shear strength was still higher than the control sample of PWH (0%). The layered pattern of PWH is attributed to the higher PF resin-filled OPA nanoparticles requirements due to the greater surface porosity and void formation developed between OPT veneer and EFB fibre mat. The maximum shear strength exhibited at 4% of PF resin-filled OPA nanoparticles is probably due to the cross-linking density occurring between PF resin, OPA nanoparticles, and layers of PWH, which results in a reduction of porosity and void content. This interaction developed between them would impede the greater shear load and hence obtained higher shear strength. A previous investigation by Candan and Akbulut [18,53,65] found that the application of SiO_2_ nanoparticles at certain particular loading enhanced the bonding strength of the plywood panels, and it seems related to the significant interaction between wood and adhesives.

In this work, the shear strength performance of PWH slightly degraded at 5% PF filled OPA nanoparticles, as observed in Figure 8a. The shear strength of plywood panels, also known as a direct indication of the bonding performance of the resin. As expected, the shear strength of PWH decreased with the increment of PF resin-filled OPA nanoparticles beyond 4% loading. The increased stress concentration could explain this behaviour at a certain part of the PWH panels due to poor dispersion of OPA nanoparticles in PF resin. The higher content of OPA nanoparticles in PF resins affected the interface bonding between PF resins and layering panels.

Moreover, numerous cracks were produced, and hence the PWH panels were incapable of resisting stress concentration. Xu et al. [19] reported that modified soy protein with SiO_2_ nanoparticles up to 1% loading enhances the bonding strength of the plywood and elimination of surface porosity. However, a further increase of SiO_2_ nanoparticles leads to the degradation of plywood panels bonding strength. Lei et al. [66] also found that the addition of sodium montmorillonite (NaMMT) nano clay into urea-formaldehyde (UF) as adhesives for plywood more than optimum loading may reduce the bonding strength and certainly a poor shear stress performance. Thus, this mechanism results in a poor load barrier, thereby decreasing the shear strength of the plywood panels. It was also observed that both PWV and PWH panels behaved slightly differently in the presence of OPA nanoparticles in PF resin. The following factors, such as PF filled OPA nanoparticles loading and type of plywood material, contributed to the shear strength significantly. It is well known that nanoparticles are prone to agglomerate in the liquid phase and increase the liquid viscosity. The performance of the liquid might be improved or deteriorated depending on the interaction between the nanomaterials and the resin or adhesives. It was reported by Roughley and Karacabeyli [67] and [68] that the consumption of nanomaterials such as nano silica is believed to be involved in the binding interaction of formaldehyde. Such interactions with active groups in formaldehyde would consequently enhance the properties of resins and thus improve the characteristics of plywood panels. Furthermore, the greater improvement can only be achieved at the optimum nanomaterial loading, while the excess of nanomaterial loading is feared to deteriorate the plywood panels.

● Impact Properties

The Izod impact test was applied in this study. The value of impact strength was calculated by the loss energy of the pendulum, the energy absorbed in breakage, and the difference of initial and final height of pendulum’s swing [69,70]. The impact strengths of PWV, PWH, and PWC are shown in Figure 8b. In Figure 8b, it is noticed that the impact strength of plywood veneer (PWV) and plywood hybrid (PWH) increased by increasing the PF filled OPA nanoparticles loading from 1% to 4%. However, after PF filled OPA nanoparticles loading exceeded 4%, the impact strength significantly decreased. The impact strength of PWC also showed comparable results to PWV and PWH with the presence of OPA nanoparticles at 4% loading. This finding indicates that the incorporation of OPA nanoparticles with PF resin produces significant results for the impact properties of plywood panels. The improvement impact resistance of PWV and PWH is probably due to the stabilization of interfacial friction stress. Certainly, this characteristic develops stronger bonding between the matrix and the panel surface. The previous study by ul Haq Bhat [70] suggested that the extra energy needed to be absorbed by the composite to break and debond the fibre and matrix.

It is observed that the PWV showed greater impact resistance compared to PWH at 4% loading of PF resin-filled OPA nanoparticles, though, at the initial improvement of impact strength, the PWH obtained greater improvement compared to PWV. At the lower loading of PF resin-filled OPA nanoparticles (1–3%) in PWH, OPA nanoparticles uniformly dispersed in the matrix and constantly spread between fibre spaces. During this process, any cavities or void between EFB mat fibre and EFB fibre/OPT veneer could be filled with or fully covered by PF resin-filled OPA nanoparticles. Then, due to vast interfacial interaction between fibre, panel surface, PF resin, and OPA nanoparticles, this reaction certainly enhanced the impact resistance of plywood panels. The energy that absorbed from the pendulum during impact test was converted into stress; then, the interaction that occurred previously acted as a bridge to propagate the stress evenly between EFB fibres and OPT veneer, resulting in increases of impact strength.

In this experiment, the impact strength of PWH slightly decreased once the PF resin-filled OPA nanoparticles exceeded 4% loading. The reduction of PWH impact resistance is probably due to the matric fracture that occurred from poor bonding between PF resin and EFB fibre/OPT veneer. At a high concentration of OPA nanoparticles in the PF resin, the agglomeration of OPA nanoparticles was highly anticipated due to their surface energy. According to Zahedi et al. [71], increasing of nanomaterials loading in the polymer could lead to stiffening of polymer chains and agglomeration. The higher content of nanomaterials caused the composites to be more fragile and certainly absorb less energy from the impact test. Therefore, a large concentration of OPA nanoparticles (5%) in PF resin could not efficiently resist fracture under stress, which weakened the impact resistance of the plywood panels.

As for the impact properties of the PWV, it is obvious that the impact strength slightly increased as the PF resin-filled OPA nanoparticles loading increased from 1% to 4%. The higher results in the impact strength were obtained at 4% of OPA nanoparticles loading with the maximum strength of 431 J/m. These results indicate that the PWV panels reinforced with OPA nanoparticles enhanced the impact properties until optimum OPA nanoparticle content was reached. The presence of OPA nanoparticles at 4% loading in the PF resin influenced the rigidity of the polymer. Furthermore, with the improvement of the PF resin, the OPA nanoparticles acted as obstacles to obstruct the crack propagation while absorbing impact energy. This means that the optimum level of PF resin-filled OPA nanoparticles at 4% loading needed more energy to past the obstacles resulting in increasing the impact strength. Moreover, introducing the OPA nanoparticles into plywood panels from the lower to optimum loading showed the existence of PF resin-OPT veneer interaction and enhancement of impact performance.

It was also observed that increasing the loading of OPA nanoparticles beyond 4%, decreased the impact strength of PWV. The resin spreading and homogenous distribution of OPA nanoparticles are the most important factors that affect the mechanical properties of the plywood panels. At the optimum loading of OPA nanoparticles, the plywood panels produced greater bonding with the cured resin and surface panels then developed rigid structural integrity. As reported by El-Bashir [72], at the higher concentration of nanomaterials in thermosetting polymer, they tended to aggregate and develop cavities in the polymer. In the case of plywood panels, an excessive aggregation of OPA nanoparticles may have created hollow spaces between OPT veneer once the PF resin cured and hardened. These hollow spaces may attribute to the lower absorption of impact energy and thus affect the impact performance of the plywood panels.

#### 3.2.5. Fracture Surface Morphology for Shear Samples

**Figure 9:**
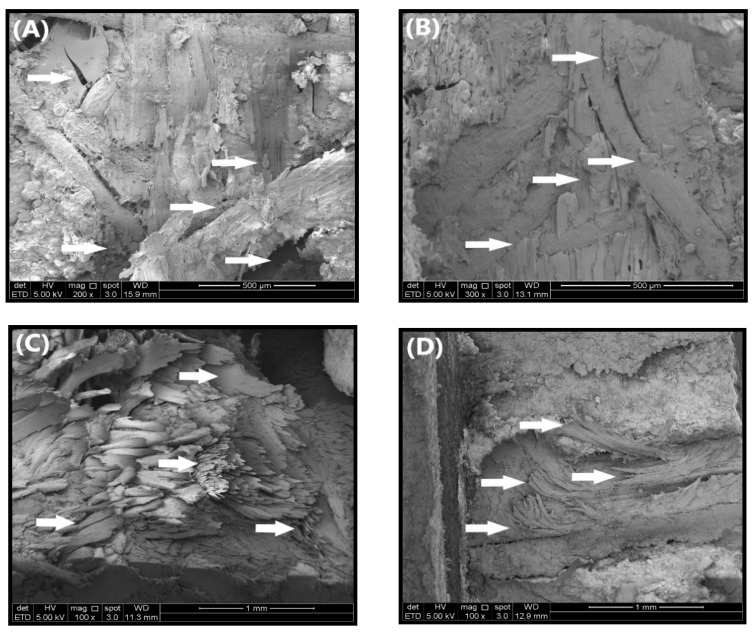
SEM micrograph of shear fracture morphology (**A**) PWH with neat PF resin; (**B**) PWH with PF resin-filled 3% OPA nanoparticles loading; (**C**) PWV with neat resin and (**D**) PWV with PF resin-filled 4% OPA nanoparticle loading.

**Figure 10:**
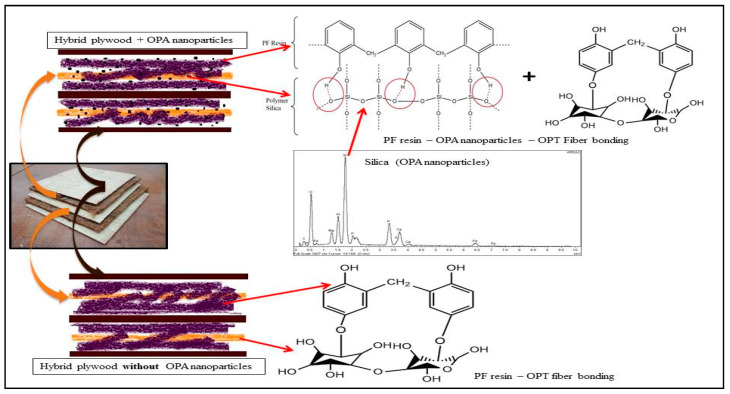
Schematic diagram showing the influence of the nanoparticles on the bonding between the layers.

The details of the fracture surface of PWH and PWV due to shear stress are presented in Figure 9A–D. From the FESEM micrograph illustrated in Figure 9, the fracture surface of fibre and veneer surface was easily recognized and identified probably due to the presence of OPA nanoparticles. Based on Figure 9A, PWH with neat PF resin shows the rough surface topography because of the reduction of stress resistance during shear stress. This is because cured and hardened neat PF resin in plywood panels creates unbonded area and porosity between the OPT veneer and EFB fibres. In many cases of shear strength reduction in plywood panels, the disrupted layers is probably due to a lack of interlaminate adhesion between OPT veneer and EFB fibres. SEM micrograph of the shear fracture surface of PWH with PF resin-filled OPA nanoparticles is illustrated in Figure 9B and indicates an improvement of shear resistance in surface fracture and better durability. From the figure, it can be explained by the fact that cured PF resin will leave the OPA nanoparticles to stick to the surface of the EFB fibre and OPT veneer. This behaviour could lead to the better capability to support stress transfer during the shear test compared to PWH with neat PF resin. The schematic chemical reactions of polymers and phenol-formaldehyde resin are shown in Figure 10.

The SEM micrograph also shows that the force did not completely break the layers, but rather separated the plies due to weak interlaminate adhesion gap. This behaviour showed that the PWH with PF resin-filled OPA nanoparticles was strong enough to tolerate the force from the shear test and prevent EFB fibre from breakage. As reported by Kumar et al. [73] who used aluminum oxide nanofiller in medium-density fibreboard (MDF), the improvement of mechanical properties was due to blocked pores of wood fibre by nanofillers, thus preventing early failure in the MDF. In another similar study, Dukarska and Czarnecki [20] also found significant improvement in shear strength properties due to enhancement of the activation energy created by nanoscopic fumed silica, ensuring a greater cross-linking process between MUPF resin and sheets of veneer.

As for PWV with neat PF resin presented in Figure 9C, the fracture occurred on the surface of the OPT veneer. This type of fracture mainly due to water that evaporated from the PF resin during the curing process before the PF resin incorporated with the unspread surface. Thus, a continuous bond line could not be formed and certainly reduced the strength of the mechanical interlock structure. In addition, the whole surface fracture appeared rough and irregular. This fracture section certainly assisted the moisture to intrude and led to low water-resistance [74]. In the meantime, from Figure 9D, it can be seen that PWV with 4% OPA nanoparticle loading showed good interface bonding between layers of plywood composites. However, it should be noted that the FESEM image clearly showed the shear resistance with the debonding of the layers after the shear test. These results demonstrate OPA nanoparticles significantly improved the interlock structure of plywood composites, which led to greater performance in shear properties.

#### 3.2.6. Fracture Surface Morphology for Impact

**Figure 11:**
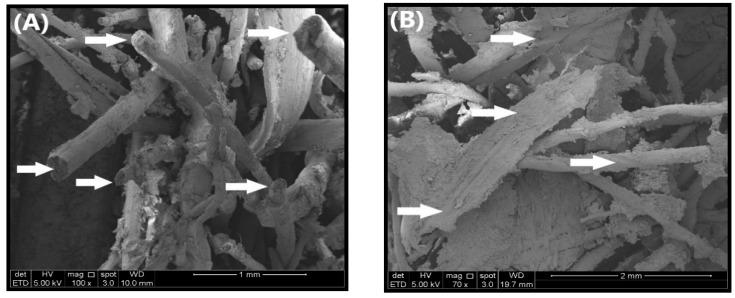

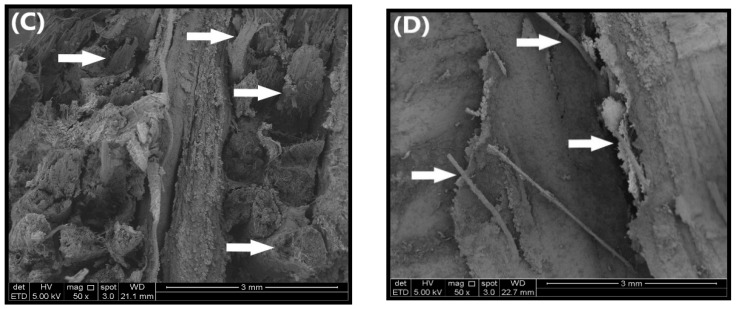
SEM micrograph of impact fracture morphology (**A**) PWH with neat PF resin; (**B**) PWH with PF resin-filled 4% OPA nanoparticles loading; (**C**) PWV with neat resin and (**D**) PWV with PF resin-filled 4% OPA nanoparticles loading.

The effect on the impact properties of plywood panels due to the presence of OPA nanoparticles was described by the Field Emission Scanning Electron Microscope (FESEM) micrograph, as shown in Figure 11. The FESEM micrograph of the impact fracture surface is taken to investigate the OPA nanoparticles/OPT fibres interaction and fracture behaviour. The interfacial interaction between OPA nanoparticles and OPT fibres is a vital factor in determining the performance of plywood panels properties. Figure 11A,B illustrated the behaviour of plywood surface morphology during the dissemination of stress in PWH with neat PF resin and PF resin-filled 4% OPA nanoparticles, respectively. The breakage of PWH with neat PF resin fibre on the impact test shown in Figure 10A was obviously due to low resistance of crack propagation. The low resistance of fibre is probably due to the properties of neat PF resin that exhibit relatively brittle behaviour after being cured and hardened. As reported by Dungani et al. [75], the properties of neat PF need to be modified or improved with nanofiller to enhance the properties, particularly in flexural and impact strength. However, the addition of nanofiller into PF resin beyond the optimum level will leads to greater fracturing due to the inefficient crack propagation from the stress applied at high speed.

In the meantime, the PWH with PF filled 4% OPA nanoparticles improved the crack propagation when force was applied at high speed, as shown in the SEM micrograph displayed in Figure 11B. As compared to PWH with neat PF resin, the fibre tended to tear apart due to stress propagation between OPA nanoparticles and the fibre wall. From the SEM micrograph, it can also be assumed that the impact force did not completely tear the OPT fibres, which seemed strong enough to tolerate the impact load due to the presence of OPA nanoparticles. Besides that, other factors such as bonding strength between PF resin-filled OPA nanoparticles and OPT fibres also exhibited a significant effect of fracture behaviour. Candan and Akbulut [53] reported that the optimum level of nanomaterial loading and nanomaterial type could lead to a synergetic effect on bonding strength and leads to the improvement of mechanical strength of nano-engineered plywood.

PWV with neat PF resin and with 4% OPA nanoparticle loading are shown in Figure 11C,D, respectively. PWV with neat PF resin in Figure 11C was critically fractured along the veneer due to lower impact resistance. It can be seen clearly that the fracture was uniformly distributed along with the plies, probably due to initial cracking by PF resin in adjacent plies. In this case, the PF matrix cracked fully in cross-section, and the stress was shared equally between fibres. Therefore, with poor crack propagation and high-stress concentration, the fracture of PWH with neat PF resin would be greater and inferior in impact resistance.

Meanwhile, the PWH with 4% OPA nanoparticles loading in Figure 11D showed a significant improvement with lower fracture failure due to better dispersion of failure mode. As reported by Zhu et al. [76], the effect of adding nanofillers at the ply interface may enhance the fracture toughness. Thus, significant improvement was considered due to the presence of OPA nanoparticles between the layers of plywood.

#### 3.2.7. Thermogravimetric (TGA-DTA) Analysis

**Figure 12:**
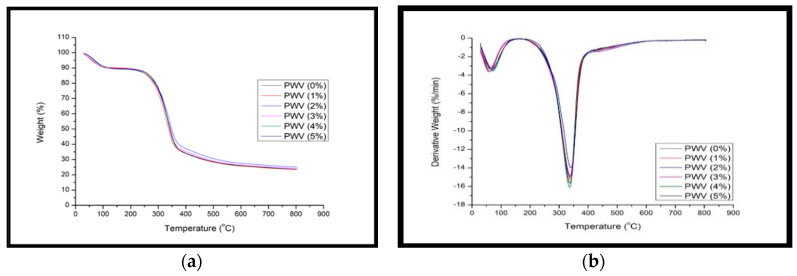
(**a**) Thermogravimetry (TGA) and (**b**) Derivative Thermogravimetric (DTG) of PWV with different loading of PF filled OPA nanoparticles.

TGA thermograms and derivative thermogravimetric curves of plywood veneer (PWV) are shown in Figure 12a,b, respectively. From the TGA curves, the presence of OPA nanofiller affected the thermal stability of the PWV composites. However, with the increase of OPA up to 5% loading, the degradation temperature of PWV was not influenced much, possibly due to cross-linking and polymerization with PF resin.

Figure 12a,b clearly show that the initial degradation of the PWV below 100 °C resulted from the gradual evaporation of moisture, previously absorbed by the hygroscopic properties of PWV. According to DTG curves in Figure 12b, the initial degradation corresponded to less than 5% mass loss in the range of 25–70 °C. The easy process of moisture evaporation from the PWV was mainly due to poor interaction of water in the PWV frame structure. This phenomenon was supported by Chaudhary et al. [77], who explained the role of water in the polymer coordination and the stress-free water dehydration process. Moreover, the peak intensity during the initial degradation showed the similarities of the dehydration process of moisture in neat and treated PWV. The similarities of the peak intensity could be associated with the same location of moisture, which occupied the void or cavities and led to effortless moisture evaporation. A study by Li et al. [73] also found that the intensity of the DTG peak represents the strength of the moisture evaporation almost at the same rate.

From the TGA and DTG curves illustrated in Figure 12a,b, respectively, it is obvious the PWV decomposed in two major steps. The major degradation occurred at the highest peak intensity at around 330–345 °C. Thermal data analysis obtained from the TGA/DTG curve is presented in Table 3. As indicated by the data analysis in Table 3, there was an improvement of the thermal stability of PWV with PF resin-filled OPA nanoparticles compared to PWV with neat PF resin. This could be confirmed with a higher residual weight obtained at the end of the degradation process and higher initial degradation temperature at the first step of the degradation process.

**Figure 13:**
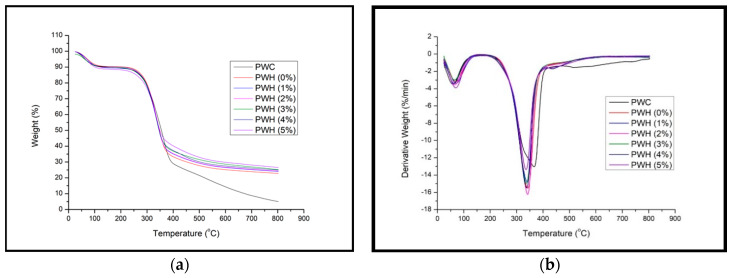
(**a**) Thermogravimetry (TGA) and (**b**) Derivative Thermogravimetric (DTG) PWV with different loading of PF filled OPA nanoparticles compared to PWC.

TGA thermograms of PWC and PWH with different loading of PF resin-filled OPA nanoparticles are illustrated in Figure 13. The effect of OPA nanoparticles on the thermostability of plywood panels was explored using TGA and DTG curves. Generally, the first stage of degradation in the 25–100 °C range involved the gradual evaporation of moisture and the light volatile content of PWH. The main degradation of PWH was from 240–410 °C and the decomposition of the PWH was complex due to multiple reactions that occurred simultaneously.

This second stage of mass loss mainly involved decomposition of the three main constituents in PWH, which are lignocellulosic materials, polymers, and nanoparticles, respectively. The major weight loss of PWH illustrated in Figure 13 mainly contributed to the degradation of lignocellulosic components. This finding also reported by Amouzgar [78] that the degradation of lignocellulosic materials was chemically active and thermochemically decomposed between 150–400 °C. In this stage, PF resin also decomposed mainly in three processes. The degradation of PF was highly dependent on its structure and cross-linking density that occurred between PF resin, OPA nanoparticles, and plywood materials. According to Chen et al. [79], the degradation of PF polymers involved an auto-oxidation process. The process included the removal of the aromatic ring structure, carbonization, and graphitization that leads to the collapse of the PF resin cure structure.

At the end of the degradation process that was recorded at 800 °C, the fraction of non-volatile matters denoted as residue remained below 30 % from the overall weight of the PWH specimens. The details of the TGA/DTG curve behaviour are presented in Table 4.

Correspondingly, at a given datum, the PWH with PF filled OPA nanoparticles was more thermally stable than the PWH with neat PF and PWC. According to Peng and Kong [80], the analysis data from the TGA/DTG curve can be used as an example to quantitatively investigate the difference of degradation temperatures between specimens. The thermostability of the specimen could be determined from the peak of degradation temperature as well as shifted to higher degradation temperature. Moreover, the higher the residual weight of the PWH compared to PWC, is also considered an improvement in thermal stability properties.

The improvement of the thermostability may be attributed to the performance of the OPA nanoparticles that could disseminate the heat from polymer onto nanoparticles. From the thermogravimetry curve illustrated in Figure 13b and analysis data presented in Table 4, the degradation of the PWH started at a higher temperature compared to PWC. The effect of thermostability by the presence of nanoparticles in nanocomposite was reported by Cui et al. [81] and Khankrua et al. [82], who found that the addition of nanoparticles protracted the degradation process and increased the thermal resistance due to high surface area of nanoparticles that assisted the heat energy to the surroundings. Though the shift of the degradation was too small or slightly towards a higher temperature range of PWH compared to PWC, this confirms the enhancement of the thermal stability of the plywood panels due to the existence of OPA nanoparticles. The main indication that could be the reason for the thermal stability could be the difference in the residual weight after 800 °C. From Table 4, it can be seen obviously that residual weight of PWH was higher compared to PWC. The difference of residual weight was due to the presence of OPA nanoparticles in PF resin that was applied onto plywood panels.

## 4. Conclusions

The results obtained from this study showed there was an improvement of plywood veneer and hybrid plywood properties such as physical, mechanical, and thermal properties compared to commercial plywood. The FT-IR analysis of PF nanocomposite filled OPA nanoparticles showed changes in the FT-IR spectrum in terms of peak intensity, peak broadening, and new absorption peak. Thermogravimetric analysis (TGA) for PF nanocomposite filled OPA nanoparticles; the PF nanocomposite exhibited better thermal stability in terms of higher initial temperature degradation, maximum temperature degradation, and percentage of ash residue. The X-ray Diffraction (XRD) results of PF nanocomposite filled OPA nanoparticles illustrated a significant presence of a peak in PF nanocomposite samples compared to neat PF. The absence of a peak in the neat PF XRD spectrum indicates that the neat PF resin consists of amorphous structures. The occurrence of a peak indicated the crystallinity material that has been incorporated with neat PF resin. The intensity of the peak in XRD spectroscopy showed the loading of OPA nanoparticles that was introduced into PF resin. Meanwhile, the micrograph of PF nanocomposite (1–5%) showed less void and porosity that was due to the incorporation of PF resin with OPA nanoparticles. This behaviour increases the viscosity and solids content of the PF resin, then leads to curing stability due to the energy of OPA nanoparticles that can disseminate heat uniformly. In density profile analysis for plywood veneer (PWV) and hybrid plywood (PWH) with PF resin-filled OPA nanoparticles, there was a significant improvement of the density profile throughout sample thickness as compared to PWV and PWH with neat PF resin and commercial plywood (PWC). The application of PF resin-filled OPA nanoparticles in plywood panel (PWV and PWH) production showed dimensional stability improvement, particularly in water absorption and thickness swelling. From the overall experimental results, the introduction of OPA nanoparticles into PF resin then applied on plywood panels showed a great improvement in terms of physical, mechanical, thermal, and fracture morphology properties. Therefore, to achieve durable and superior properties performance, proper addition of OPA nanoparticles can be introduced into plywood panels.

## Figures and Tables

**Table 1:** Ball milling process parameters.

Parameter	Level	Description
Milling Time	3	24 h
Milling Speed	1	100 rpm
Size of balls	3	20 mm

**Table 2:** Specification of oil palm trunk (OPT) veneer and empty fruit bunch (EFB) fibre mat.

Materials	Size (mm)	Thickness (mm)	Moisture Content (%)
OPT Veneer	300 × 300	4	12–15 [30,31]
EFB Fibre Mat	300 × 300	6	10–12 [31,32]

**Table 3:** Thermal parameters for the thermograms of PWV with different loading of PF resin-filled OPA nanoparticles.

Type of Plywood	Initial Degradation Temperature (°C)	Maximum Degradation Temperature (°C)	Residue (%)
PWV 0%	250	330	23
PWV 1%	255	339	24
PWV 2%	259	340.	25
PWV 3%	253	337	24
PWV 4%	254	338	26
PWV 5%	254	339	26

**Table 4:** Thermal parameters for the thermograms of PWH and PWC.

Type of Plywood	Initial Degradation Temperature (°C)	Maximum Degradation Temperature (°C)	Residue (%)
PWC	240	330	5
PWH 0%	267	335	25
PWH 1%	269	339	24
PWH 2%	280	337	24
PWH 3%	284	366	25
PWH 4%	279	340	27
PWH 5%	279	342	25
